# Clonal Hematopoiesis of Indeterminate Potential and Atrial Fibrillation: Insights into Pathophysiology and Clinical Implications

**DOI:** 10.3390/ijms26062739

**Published:** 2025-03-18

**Authors:** Paschalis Karakasis, Panagiotis Theofilis, Eleftheria Lefkou, Antonios P. Antoniadis, Dimitrios Patoulias, Panagiotis Korantzopoulos, Nikolaos Fragakis

**Affiliations:** 1 Second Department of Cardiology, Hippokration General Hospital, Medical School, Aristotle University of Thessaloniki, Konstantinoupoleos 49, 54642 Thessaloniki, Greece; aantoniadis@gmail.com (A.P.A.); fragakis.nikos@googlemail.com (N.F.); 2 First Cardiology Department, School of Medicine, Hippokration General Hospital, National and Kapodistrian University of Athens, 11527 Athens, Greece; panos.theofilis@hotmail.com; 3 Perigenesis, Institute of Obstetric Haematology, 54623 Thessaloniki, Greece; elefkou@gmail.com; 4 Second Propedeutic Department of Internal Medicine, Faculty of Medicine, Aristotle University of Thessaloniki, 54642 Thessaloniki, Greece; dipatoulias@gmail.com; 5 First Department of Cardiology, School of Health Sciences, Faculty of Medicine, University of Ioannina, 45500 Ioannina, Greece; pkorantz@uoi.gr

**Keywords:** clonal hematopoiesis of indeterminate potential, DNMT3A, TET2, ASXL1, atrial fibrillation, inflammation, atrial fibrosis

## Abstract

Clonal hematopoiesis of indeterminate potential (CHIP) has emerged as a novel risk factor for cardiovascular diseases. CHIP is characterized by the expansion of hematopoietic stem cell clones harboring somatic mutations in genes such as TET2, DNMT3A, and ASXL1, which are implicated in inflammation, atrial remodeling, and hypercoagulability. These mutations foster a pro-inflammatory and pro-thrombotic environment conducive to arrhythmogenesis, thereby linking CHIP to the development and progression of atrial fibrillation (AF). Mechanistic insights indicate that CHIP contributes to atrial fibrosis, disrupts calcium signaling, and exacerbates oxidative stress, all of which heighten susceptibility to AF. Clinical studies, including epidemiological and Mendelian randomization analyses, further support the association between CHIP and an increased risk of both incident and progressive AF, with specific mutations such as TET2 and ASXL1 identified as significant contributors. Additionally, CHIP has been linked to adverse outcomes in AF, including elevated rates of heart failure, thromboembolism, and mortality. Understanding CHIP’s role in AF pathophysiology offers opportunities for the development of precision medicine approaches, providing novel avenues for early intervention and targeted AF treatment. This review synthesizes current mechanistic and clinical evidence on the role of CHIP in AF, emphasizes its potential as a biomarker for risk stratification, and explores emerging therapeutic strategies targeting CHIP-associated pathways.

## 1. Introduction

Atrial fibrillation (AF), the most prevalent sustained cardiac arrhythmia, affects approximately 2–4% of the global adult population and is strongly associated with significant morbidity and mortality [1,2,3,4,5]. The increasing prevalence of AF with age underscores its substantial impact on healthcare systems worldwide [4,6,7]. AF contributes to a heightened risk of ischemic stroke, heart failure, and premature death, presenting both clinical and economic challenges [5,8,9]. The pathophysiology of AF is complex, involving structural, electrical, and mechanical remodeling of the atrial tissue, often exacerbated by systemic inflammation and oxidative stress [10,11,12,13,14].

As individuals age, the accumulation of genetic mutations within somatic cells gives rise to genomic alterations not present in the germline, a phenomenon known as somatic mosaicism [15]. Hematopoietic stem cells (HSCs), due to their high proliferative capacity, are particularly susceptible to the development of such mosaicism [15]. When a mutated clone gains a selective advantage, it can expand and contribute significantly to the peripheral blood cell population, a process referred to as clonal hematopoiesis [16]. Clonal hematopoiesis of indeterminate potential (CHIP) is defined by the acquisition of somatic mutations in HSCs, leading to clonal expansion in the absence of overt hematologic malignancy [17,18]. Frequently associated with advancing age, CHIP affects up to 10–20% of individuals over the age of 70 [19,20]. The most commonly implicated genes in CHIP include DNA methyltransferase 3A (DNMT3A), ten-eleven translocation methylcytosine dioxygenase 2 (TET2), and Additional sex combs-like 1 (ASXL1), which are involved in epigenetic regulation, mRNA splicing, and DNA damage repair [20,21]. Although CHIP has been recognized for decades, the advent of large-scale exome sequencing has only recently facilitated comprehensive investigations into its prevalence, longitudinal clinical implications, and gene-specific associations in large populations. Initially identified in the context of hematological disorders, CHIP has more recently been implicated as a key contributor to cardiovascular pathology [22,23]. Of note, emerging evidence suggests that CHIP plays a significant role in the pathogenesis of various cardiovascular diseases, including myocardial infarction [24,25], ischemic stroke [26], and heart failure [27,28,29,30,31,32,33,34,35,36,37,38,39].

Recent evidence suggests that CHIP may also play a pivotal role in the development and progression of AF [35,40]. Mechanistic studies have demonstrated that mutations in genes such as TET2 and DNMT3A drive the production of pro-inflammatory cytokines, including interleukin (IL)-1 and IL-6 [41]. These cytokines promote atrial remodeling and fibrosis, disrupt calcium signaling, and contribute to the creation of a pro-arrhythmic substrate. CHIP is also implicated in the amplification of oxidative stress and endothelial dysfunction, further exacerbating the susceptibility of atrial tissue to arrhythmogenesis [42,43]. Furthermore, CHIP has been associated with hypercoagulability, which may further increase the risk of thromboembolic complications in AF, including ischemic stroke and systemic embolism [44,45,46].

Epidemiological data corroborate the association between CHIP and AF. Population-based studies, including analyses from the UK Biobank, have revealed an elevated prevalence of CHIP among individuals with AF, independent of conventional risk factors such as age, hypertension, and diabetes [35]. Gene-specific analyses indicate that mutations in TET2 confer the highest relative risk for AF, suggesting distinct mechanistic contributions of specific CHIP mutations [40,41]. In addition, the presence of CHIP has been associated with adverse clinical outcomes in patients with AF, including increased rates of heart failure, ischemic stroke, and mortality [40].

With the growing accessibility of affordable next-generation sequencing and the advent of clinical trials exploring CHIP-directed therapeutics [47], the potential to identify conditions amenable to CHIP-specific prevention or intervention strategies has become increasingly actionable. The integration of CHIP into the broader understanding of AF pathophysiology offers a novel framework for exploring its mechanisms and therapeutic implications. The pro-inflammatory and pro-thrombotic milieu created by CHIP represents a convergence of biological pathways that may drive arrhythmogenesis and associated complications. Consequently, targeting these pathways through CHIP-specific interventions could present new opportunities for improving outcomes in patients with AF.

This review explores the intersection of CHIP and AF, highlighting mechanistic insights, clinical evidence, and therapeutic opportunities. By synthesizing data from basic science, clinical studies, and population research, it aims to clarify CHIP’s role in AF and its implications for clinical practice and future research.

## 2. Mechanistic Insights

### 2.1. CHIP, Inflammation and Atrial Remodeling

**Figure 1:**
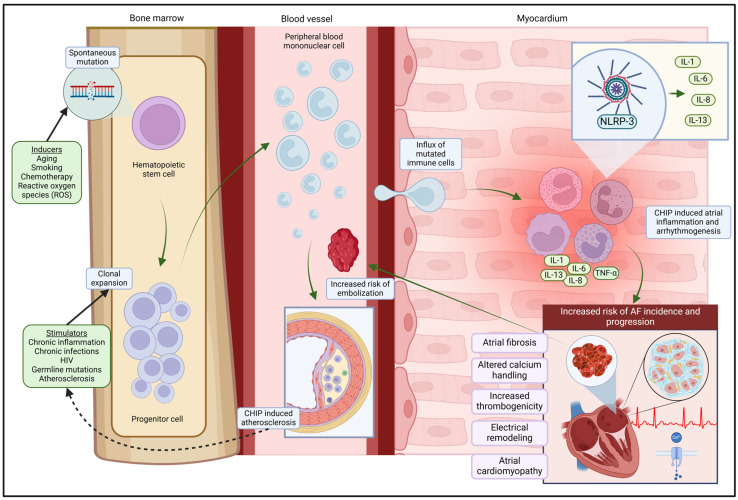
The association between clonal hematopoiesis of intermediate potential (CHIP) and atrial fibrillation (AF). Mutations occurring in hematopoietic stem and progenitor cells result in the formation of clonal populations that expand progressively over time. Various factors contribute to the stimulation of clonal proliferation. As a consequence, these mutated cells infiltrate the bloodstream and myocardium, promoting atherosclerosis and adversely affecting cardiac function. A pivotal mechanism underlying clonal hematopoiesis-induced atrial inflammation and arrhythmogenesis involves an inflammasome-mediated response, particularly through the interleukin-1/interleukin-6 signaling axis. Abbreviations: CHIP, clonal hematopoiesis of indeterminate potential; IL-1, interleukin-1; IL-6, interleukin-6; IL-8, interleukin-8; IL-13, interleukin-13; NLRP3, NLR family pyrin domain-containing 3; ROS, reactive oxygen species; TNF-α, tumor necrosis factor-alpha.

The relationship between CHIP and AF appears to be primarily driven by heightened inflammatory and immune activity (Figure 1). Somatic mutations in TET2 are known to amplify inflammation within cardiac macrophages, resulting in increased production of pro-inflammatory mediators such as IL-1, IL-6, IL-8, and atherogenic chemokines [24,48]. Similarly, DNMT3A-deficient mast cells demonstrate hyperactivity and enhanced secretion of cytokines, including IL-6, tumor necrosis factor (TNF), and IL-13, following immunological stimulation [49]. The role of inflammation in AF pathophysiology is well documented, with histological evidence of lymphomononuclear cell infiltration and adjacent myocyte necrosis [50], alongside elevated levels of systemic inflammatory markers, such as C-reactive protein (CRP), heat shock proteins, IL-6, IL-8, and TNF [14,51,52]. Elevated high-sensitivity CRP (hs-CRP) levels in individuals with both CHIP and AF further underscore a shared inflammatory axis [40]. Greater inflammatory responses were observed in CHIP carriers than in non-carriers both pre- and postoperatively [53]. Moreover, statistical analyses reveal that the association between CHIP and AF diminishes when hs-CRP levels are considered, strongly suggesting that inflammation mediates this relationship [40].

Emerging evidence suggests that CHIP-associated mutations contribute to cardiovascular pathology through distinct inflammatory pathways. A recent study utilizing a CRISPR/Cas9-based lentiviral system introduced inactivating mutations in Tet2 and Dnmt3a within hematopoietic stem/progenitor cells, which were subsequently engrafted into lethally irradiated mice [54]. Upon angiotensin II (Ang II) infusion, both Tet2- and Dnmt3a-mutant mice exhibited exacerbated cardiac hypertrophy, impaired cardiac function, and increased cardiac and renal fibrosis, reinforcing the role of CHIP in cardiovascular dysfunction [54]. Importantly, the study identified mutation-specific inflammatory signatures. Tet2 deficiency was associated with upregulation of IL-1β, IL-6, and Ccl5, while Dnmt3a inactivation led to increased expression of Cxcl1 (CXC chemokine ligand), Cxcl2, IL-6, and Ccl5 in lipopolysaccharide-stimulated macrophages [54]. These findings highlight distinct inflammatory pathways through which CHIP mutations may contribute to cardiovascular disease, suggesting that while both Tet2 and Dnmt3a mutations promote inflammation-driven pathology, they do so via differential molecular mechanisms.

The pro-inflammatory state induced by CHIP-associated mutations is hypothesized to contribute to atrial remodeling through structural, electrophysiological, and autonomic pathways, thereby promoting the initiation and progression of AF [35]. CHIP carriers exhibit elevated levels of activated circulating monocytes and monocyte-derived macrophages with inflammatory gene expression profiles, which likely amplify systemic and local inflammatory responses [53].

Additionally, CHIP has been linked to increased myocardial fibrosis, as reflected by elevated T1 times in cohort studies and experimental models involving TET2 mutations [35,48,55,56,57]. Myocardial interstitial fibrosis is implicated as a critical factor for AF, with fibrotic alterations in the myocardium known to disrupt electrical impulse conduction and facilitate reentrant circuits, predisposing individuals to AF [35,58,59]. Both clinical and preclinical evidence support these findings; overexpression of the NOD-like receptor family pyrin domain containing 3 (NLRP3) inflammasome, observed in CHIP patients [41], within cardiomyocytes has been shown to increase atrial fibrosis and susceptibility to AF, underscoring the role of inflammation-induced fibrotic remodeling in arrhythmogenesis [60,61,62]. Moreover, recent studies highlight the role of monocyte/macrophage polarization in atrial fibrosis and arrhythmogenesis, with CHIP mutations influencing the balance between pro-inflammatory M1 and reparative M2 macrophage subsets [63]. TET2 loss-of-function mutations have been shown to promote an M1-dominant profile, characterized by elevated IL-1β and IL-6 expression, exacerbating atrial fibrotic remodeling [64,65]. Single-cell transcriptomic analyses further support this shift in myeloid cell polarization [63], demonstrating increased inflammatory macrophage infiltration in CHIP carriers, which may contribute to profibrotic signaling and conduction abnormalities.

Fibrotic remodeling associated with CHIP also contributes to an increased risk of heart failure [27,28,29,30,31,32,33,34,35,36,37,38,39], which constitutes an additional independent factor for the incidence and perpetuation of AF [66]. Collectively, these findings underscore the central role of dysregulated inflammation and fibrosis in bridging CHIP and AF pathophysiology.

### 2.2. CHIP and Altered Calcium Handling

The disruption of calcium homeostasis is a critical mechanism linking CHIP to AF. Loss of TET2, a gene frequently mutated in CHIP, has been shown to impair calcium handling in cardiomyocytes through mechanisms involving the NLRP3 inflammasome [41]. Hematopoietic-specific inactivation of TET2 in murine models leads to increased activation of CaMKII (Ca^2+^/calmodulin-dependent protein kinase II), a key regulator of calcium flux within the sarcoplasmic reticulum (SR) [41]. This dysregulation is known to promote aberrant calcium release from the SR into the cytosol, promoting atrial arrhythmogenesis [67,68,69,70]. Specifically, cardiomyocytes from TET2-deficient mice exhibit impaired calcium transient dynamics, including prolonged time to peak and an increased frequency of spontaneous calcium release events [60].

Prior research has highlighted the critical role of cardiac macrophages in modulating electrical conduction and promoting AF in murine models, potentially through mechanisms involving direct intercellular communication and paracrine signaling pathways that influence cardiac conduction and susceptibility to AF [71,72,73,74]. Experimental studies demonstrate that TET2-deficient macrophages can exacerbate calcium dysregulation through paracrine effects, highlighting the role of inflammatory mediators such as IL-1β and IL-6 in altering SR calcium release [41]. Of note, in vitro models of human atrial cardiomyocytes co-cultured with TET2-deficient macrophages show reduced SR calcium content and decreased transient amplitudes, mirroring observations in murine cardiomyocytes [41]. This altered calcium handling contributes to electrical remodeling of the atrial substrate, characterized by shortened action potential duration and increased AF susceptibility [41]. However, further investigation is imperative to elucidate the molecular mechanisms underpinning the interplay between NLRP3 activation, CaMKII signaling, and SR calcium release, as well as to delineate the contributions of TET2 deficiency in macrophages to these pathophysiological alterations.

### 2.3. CHIP and Thrombogenic Potential

CHIP is closely associated with a pro-thrombotic state, potentially influencing the risk of thromboembolic complications in AF [75]. CHIP-associated mutations, particularly in JAK2, are strongly implicated in enhanced thrombotic risk [76,77]. Janus kinase 2 (JAK2) mutations contribute to increased megakaryocyte activity, heightened platelet reactivity via hypersensitivity of the thrombopoietin receptor (MPL), and elevated levels of procoagulant microvesicles [76,78]. These changes amplify the coagulation cascade, promoting thrombus formation [79].

In addition to JAK2, mutations in TET2 and DNMT3A play critical roles in CHIP-mediated hypercoagulability [80]. TET2 mutations drive the upregulation of inflammatory cytokines, such as IL-1β and IL-6, which further exacerbate endothelial dysfunction and thrombin generation [80]. The resulting pro-inflammatory state synergizes with coagulative pathways, creating an environment that predisposes individuals with CHIP to thromboembolic events. Clinical evidence supports these findings, demonstrating a higher incidence of thrombotic complications, including stroke and systemic embolism, among individuals with CHIP mutations, independent of traditional cardiovascular risk factors [26,81,82,83].

## 3. Clinical Evidence

Although the findings are still preliminary, there is a growing body of evidence from cohort studies and Mendelian randomization analyses supporting an association between CHIP and AF, suggesting its potential relevance in disease pathogenesis and perpetuation.

An East Asian cohort study investigated the association between CHIP and AF, along with its clinical implications for AF progression and related outcomes [40]. The study included 1004 patients with AF and 3341 healthy controls without AF. CHIP was identified using deep-targeted sequencing with a mean coverage depth of 1000×, focusing on 24 CHIP-associated genes. A variant allele fraction (VAF) threshold of ≥2% was used to define CHIP. Multivariable logistic regression models were applied to assess the prevalence of CHIP in AF patients, its association with clinical features, and its impact on outcomes [40]. For validation, data from the UK Biobank, including 21,286 AF patients, were analyzed to explore the risk of a composite outcome comprising heart failure HF, ischemic stroke IS, and death [40].

The findings revealed that CHIP mutations were significantly more prevalent in AF patients (23.6%) than in controls (10.7%), with adjusted odds ratios (OR) of 1.38 for all CHIP mutations and 1.65 for TET2 mutations [40]. Gene-specific analyses identified TET2, DNMT3A, and ASXL1 as the most common mutations, with TET2 showing the strongest association with AF [40]. AF patients harboring CHIP mutations exhibited worse clinical features, including older age, longer AF duration, greater left atrial enlargement, higher E/E’ values indicative of diastolic dysfunction, and an increased prevalence of diabetes [40]. Notably, TET2 mutations were strongly associated with severe left atrial remodeling and prolonged AF duration. Importantly, in the UK Biobank cohort, AF patients with CHIP mutations had a 1.32-fold higher risk of the composite outcome of HF, IS, and death, primarily driven by a 1.27-fold increased risk of HF and a 1.54-fold higher risk of death [40]. The CHIP-associated risk remained significant after adjusting for potential confounders, including age, sex, and cardiovascular comorbidities [40].

Schuermans et al. conducted a large-scale study leveraging data from the UK Biobank to investigate the relationship between CHIP and incident arrhythmias, with a specific focus on AF [35]. The cohort included over 410,000 middle-aged adults, all of whom were free of arrhythmias at baseline. Clonal hematopoiesis was identified using whole-exome sequencing, with VAF of ≥2% (any CHIP) and ≥10% (large CHIP) serving as primary exposures [35]. The study incorporated multivariable-adjusted Cox regression models to evaluate the associations of CHIP with arrhythmias and to control for confounding factors such as coronary artery disease, heart failure, and demographic variables. The median follow-up period was 11.1 years, providing robust longitudinal data on arrhythmic events [35].

CHIP was found to be independently associated with an increased risk of multiple arrhythmia subtypes, including supraventricular arrhythmias (HR 1.11), bradyarrhythmias (HR 1.09), and ventricular arrhythmias (HR 1.16) for VAF ≥ 2%, with stronger associations observed for VAF ≥ 10% [35]. Secondary analyses revealed significant associations with AF (HR 1.11) and cardiac arrest (HR 1.29), underscoring CHIP’s role in atrial arrhythmogenesis. Importantly, gene-specific analyses highlighted mutations in TET2, ASXL1, and spliceosome-related genes as being particularly associated with an increased risk of arrhythmias [35].

The investigators also demonstrated that CHIP-associated mutations promote systemic inflammation and myocardial remodeling, which may predispose individuals to the development of AF [35]. Participants with large CHIP clones had a significantly higher cumulative incidence of AF compared to those without CHIP, and these associations persisted even after adjusting for other cardiovascular risk factors [35]. Elevated T1 times in individuals with TET2 mutations point to a mechanistic link between CHIP-related fibrosis and arrhythmogenic atrial substrates [35]. Furthermore, the study identified a gene-specific predisposition, with TET2 mutations conferring higher risks of AF and cardiac arrest compared to DNMT3A mutations [35].

Another study by Lin et al. combined data from clinical and murine models to explore the role of CHIP, particularly TET2 mutations, in the pathogenesis of AF [41]. In this large cohort, encompassing over 358,000 participants, CHIP was identified using whole-exome sequencing with a VAF threshold of ≥2%. Incident AF was assessed using Cox proportional hazard models, adjusting for key covariates such as age, body mass index, and comorbidities [41]. Murine models, including mice with hematopoietic-specific inactivation of Tet2, were employed to elucidate the mechanistic underpinnings of CHIP in AF development, focusing on the NLRP3 inflammasome and calcium handling in cardiomyocytes [41].

The findings demonstrated that CHIP was associated with a significantly increased risk of incident AF (HR 1.11), with TET2 mutations conferring the highest relative risk compared to other CHIP-associated mutations [41]. Larger clone sizes (VAF ≥ 10%) were linked to an even greater risk of AF. In murine models, hematopoietic-specific loss of Tet2 increased AF susceptibility through activation of the NLRP3 inflammasome and subsequent calcium-handling abnormalities in atrial cardiomyocytes [41]. Key findings included shortened atrial effective refractory periods, elevated phosphorylated CaMKII levels, and disrupted sarcoplasmic reticulum calcium release [41]. These changes were mediated by Tet2-deficient macrophages, which amplified inflammatory signaling via cytokines IL-1β and IL-6, further exacerbating arrhythmogenesis [41]. Pharmacological inhibition of the NLRP3 inflammasome with NP3-361 effectively mitigated these effects, reducing AF susceptibility in Tet2 knockout mice [41].

A population-based, prospective cohort study by Saadatagah et al. [84] investigated the association between CHIP, AF, inflammatory biomarkers, and cardiac remodeling. The study analyzed data from two cohorts: the Atherosclerosis Risk in Communities (ARIC) study and the UK Biobank. A total of 199,982 participants were included, with 4131 participants from the ARIC cohort (mean age 76 years, 59% female, 23% Black) and 195,851 participants from the UK Biobank cohort (mean age 56 years, 55% female, 94% White) [84]. The median follow-up was 7 years for ARIC and 12.2 years for UKB participants. CHIP was identified in 24.7% of ARIC participants and 5.8% of UKB participants, with large CHIP observed in 11.6% and 2.6%, respectively [84].

Meta-analysis demonstrated that large CHIP was associated with an increased risk of incident AF (HR 1.12) [84]. Gene-specific analyses revealed that large TET2 CHIP was linked to a 29% increased risk of AF (HR 1.29), while large ASXL1 CHIP conferred a 45% increased risk (HR 1.45) [84]. These associations were not observed for DNMT3A. Mechanistically, large TET2 CHIP was associated with elevated IL-6 levels, indicating a pro-inflammatory state, while large ASXL1 CHIP was linked to increased high-sensitivity troponin T (hs-TnT) levels and higher left ventricular mass index, suggesting significant cardiac remodeling [84].

Regan et al. explored the association between CHIP and AF in a high-risk cohort of 8469 participants referred for cardiac catheterization [85]. The prevalence of CHIP in the cohort was 5.0%, with DNMT3A, TET2, and ASXL1 being the most commonly mutated genes [85]. Large CHIP clones (VAF ≥ 10%) were identified in 3.2% of participants. While the study observed a significant association between CHIP and higher odds of prevalent AF in univariate models (OR 1.72), these findings did not remain significant after adjustment for covariates, including age, sex, and comorbidities [85]. However, gene-specific analyses revealed that ASXL1 CHIP was associated with a 2.15-fold increased risk of incident AF in multivariate models, highlighting its potential role in arrhythmogenesis [85]. Taken together, these results suggest that specific CHIP mutations, such as ASXL1, may contribute to AF development, although the overall association of CHIP with AF requires further investigation.

### Evidence from Mendelian Randomization

Mendelian randomization (MR) offers a robust framework for establishing causal relationships by leveraging genetic variants as instrumental variables to mitigate confounding and reverse causality. In a recent study by Kar et al., genetic liability to CHIP was assessed in a cohort of over 200,000 participants [86]. The analysis revealed a significant causal relationship between CHIP and AF, with genetically predicted CHIP increasing AF risk (odds ratio [OR]: 1.09; 95% CI: 1.04–1.15). This association was consistent across CHIP driven by DNMT3A mutations, as well as large clones with a VAF ≥ 10% [86]. These findings complement prior observational data and underscore the role of CHIP in AF development, likely mediated by pathways involving inflammation and atrial remodeling. However, MR has inherent limitations, including pleiotropy, where genetic variants may independently influence AF risk, and confounding by age-related immune dysregulation and comorbidities such as hypertension and diabetes [87,88]. While MR provides valuable insights, its findings should be interpreted cautiously and complemented by mechanistic studies to establish a definitive causal link [87,88]. A summary of studies investigating the association between CHIP and AF is presented in Table 1.

## 4. Clinical Implications of CHIP in the Management of AF

The identification of CHIP as a risk factor for AF may have relevant clinical implications. CHIP-associated mutations, particularly in genes such as TET2, ASXL1, and JAK2, contribute to atrial remodeling, systemic inflammation, and thrombogenicity, which are central to AF pathogenesis [12,89,90]. These mechanisms suggest that CHIP testing could enhance current risk stratification models for AF, particularly in patients with unexplained or nontraditional risk factors [91]. Furthermore, the pro-inflammatory and hypercoagulable state linked to CHIP mutations underscores the potential for targeted therapeutic strategies, such as anti-inflammatory agents or novel anticoagulants, tailored to CHIP-positive individuals.

AF is widely recognized as a chronic and progressive disorder, typically initiating in a paroxysmal form and advancing to more sustained forms, such as persistent or long-standing persistent AF [92]. This progression is primarily driven by atrial remodeling, characterized by structural and functional alterations, including left atrial enlargement and increased LA stiffness [93,94]. Specifically, the observed association between CHIP and atrial fibrillation progression has significant clinical implications for risk stratification, early intervention, and therapeutic management. CHIP mutations, particularly in genes like TET2 and DNMT3A, are linked to advanced atrial remodeling, including left atrial enlargement, increased stiffness, and prolonged AF duration, which contribute to the transition from paroxysmal to persistent or long-standing persistent AF [40]. This progression is known to be associated with worsened cardiovascular outcomes, including higher risks of heart failure, thromboembolism, and mortality [95,96].

The relationship between CHIP and AF has been increasingly recognized, with several studies highlighting the distinct roles of specific mutations. Mutations in TET2 and ASXL1 have been consistently associated with an elevated risk of AF [40,41,85], suggesting that these genetic alterations contribute to atrial remodeling and arrhythmogenesis through pro-inflammatory and fibrotic mechanisms. Conversely, while DNMT3A mutations are among the most frequently observed in CHIP, current evidence does not support a direct correlation between DNMT3A mutations and AF-related pathology [40,41]. The differential effects of these mutations may underscore the importance of gene-specific analyses in understanding the mechanistic underpinnings of CHIP-associated AF.

Recognizing CHIP as a risk factor for AF progression emphasizes its potential role as a biomarker for identifying patients at higher risk of severe disease forms and complications. Early detection of CHIP mutations could enable clinicians to implement targeted monitoring and interventions aimed at slowing AF progression, such as advanced imaging to assess atrial remodeling, more aggressive rhythm control strategies, earlier referral for catheter ablation, and optimized anticoagulation to mitigate thromboembolic risk. Furthermore, understanding the dose–response relationship between CHIP VAF and AF duration may allow for personalized treatment approaches.

Existing AF risk prediction models do not incorporate CHIP status, and its prognostic significance relative to traditional risk factors remains unclear [91]. The development and validation of CHIP-specific AF risk models in prospective cohorts will be essential to determining its incremental value in risk stratification. Moreover, while CHIP-driven inflammation and thrombogenicity have been implicated in atrial remodeling and arrhythmogenesis, the extent to which CHIP constitutes a modifiable risk factor remains undetermined. Future research should investigate whether optimizing cardiovascular health can attenuate the impact of CHIP on AF incidence and progression. Future studies should explore the role of CHIP profiling in refining AF management algorithms, with particular emphasis on mutation-specific effects and their implications for personalized medicine.

## 5. Therapeutic Targeting of CHIP in AF

With increasing evidence suggesting that pro-inflammatory cytokines serve as a critical biological link between clonal hematopoiesis and AF, it is reasonable to hypothesize that mitigating inflammation through targeted interventions within the CHIP-inflammation axis may reduce the risk of incident AF and its progression.

Inflammasome activity represents a promising therapeutic target, with potential modulation through specific inhibitors of NLRP3 or AIM2. Several compounds aimed at inhibiting NLRP3 deubiquitination are currently under development. Among these, thiolutin, a zinc chelator, has demonstrated efficacy in suppressing IL-1 beta production by inhibiting NLRP3 deubiquitination. A phase 1c multicenter, randomized clinical trial (GC43343) is presently evaluating the safety of NLRP3 inhibition with selnoflast in patients with CAD and elevated hs-CRP levels [97]. Notably, a substudy within this trial focuses on individuals harboring pathogenic TET2 CHIP mutations. While the primary objective is to assess the safety profile of selnoflast, secondary endpoints (hs-CRP) and exploratory endpoints (IL-1 beta) aim to elucidate its effects on systemic and CHIP-associated inflammation. Importantly, NLRP3 inhibition is anticipated to exhibit a reduced immunosuppressive profile compared to anti-IL-1 beta therapies, as IL-1 beta is also produced by other inflammasomes. Similarly, inhibition of the AIM2 inflammasome offers potential for AF risk reduction, with preliminary studies in murine models demonstrating significant decreases in inflammatory burden following treatment with AIM2-antagonizing synthetic oligonucleotides [98].

Colchicine, which exhibits inhibitory effects on NLRP3 inflammasome activity and IL-1β, has demonstrated efficacy in secondary prevention of cardiovascular events [99]. Similarly, the role of IL-6 in cardiovascular disease is being actively investigated in ongoing clinical trials evaluating anti–IL-6 therapies in patients with coronary artery disease and chronic kidney disease [100]. In the CANTOS (Canakinumab Anti-Inflammatory Thrombosis Outcomes Study) trial, treatment with the IL-1β neutralizing monoclonal antibody canakinumab significantly reduced major adverse cardiac events and heart failure hospitalizations in patients with established CAD and elevated high-sensitivity C-reactive protein (hsCRP). Notably, post hoc analyses revealed that individuals harboring TET2 CHIP mutations experienced greater clinical benefit from canakinumab compared to non-CHIP carriers [30]. These findings highlight the potential for somatic mutation-guided, targeted anti-inflammatory therapies and warrant further investigation to explore their implications in context of AF.

An alternative therapeutic approach focuses on targeting cellular mediators downstream of inflammasomes, leveraging their involvement in signaling pathways implicated in the development and progression of AF. For instance, monoclonal antibodies against IL-6, such as ziltivekimab, are under development with the aim of mitigating cardiovascular disease risk [101]. Similarly, pharmacological inhibition of inflammatory cytokines elevated in individuals with CHIP, such as IL-1 beta (anakinra) and IL-18 (tadekinig alfa), holds promise for attenuating AF onset and progression.

A third class of therapeutic interventions focuses on CHIP mutation-specific strategies. Hypomethylating agents, such as azacytidine and decitabine, have demonstrated efficacy in treating individuals with TET2-mutant myeloid malignancies and warrant investigation for their potential to prevent AF in patients with CHIP. Vitamin C has been shown to mimic TET2 restoration by enhancing tumor sensitivity to DNA damage and suppressing leukemia progression in TET2-deficient mouse hematopoietic stem and progenitor cells [102]. Similarly, JAK2 inhibitors present a targeted approach for addressing JAK2 V617F mutations. For example, ruxolitinib, a Janus kinase 1 (JAK1) and JAK2 inhibitor, has demonstrated anti-inflammatory effects in murine models, while fedratinib, a selective JAK2 inhibitor, offers potential advantages by minimizing off-target effects [103]. Additionally, therapies targeting mutations in splicing factors (e.g., SF3B1 modulators) and mutant tumor protein P53 (TP53) represent promising avenues for addressing CHIP-associated residual inflammatory risk.

Finally, another promising class of agents with potential antifibrotic properties is sodium-glucose co-transporter 2 (SGLT2) inhibitors and glucagon-like peptide-1 receptor agonists (GLP-1RAs). These drugs have demonstrated significant cardio-renal-metabolic benefits, with emerging evidence suggesting their effectiveness in reducing AF incidence and progression, regardless of diabetes status [104,105,106,107,108,109,110,111,112,113,114,115,116,117,118,119,120,121,122,123]. SGLT2 inhibitors exert antifibrotic effects through multiple mechanisms. By enhancing mitochondrial function and reducing reactive oxygen species production [124,125], they mitigate oxidative stress and suppress pro-fibrotic signaling pathways, including the TGF-β/SMAD axis [126]. Furthermore, SGLT2 inhibitors exhibit anti-inflammatory properties and may attenuate CHIP-induced inflammation by reducing circulating levels of cytokines such as IL-6 and TNF-α, which are key contributors to cardiac fibroblast activation and extracellular matrix remodeling [127,128,129]. On the other hand, GLP-1RAs directly counteract atrial fibrosis by modulating cardiac fibroblast activity through GLP-1 receptor activation [130,131] and Indirectly, alleviate cardiac hypertrophy, lessening mechanical strain on atrial tissue and further mitigating fibrosis [132,133]. Additionally, GLP-1RAs significantly lower systemic and local inflammation by attenuating macrophage infiltration and pro-inflammatory cytokine levels [134,135]. However, further research is needed to solidify the therapeutic potential of SGLT2 inhibitors and GLP-1RAs in the context of CHIP-associated AF.

## 6. Future Directions

Despite significant advancements in understanding the relationship between CHIP and AF, several limitations in current research highlight critical areas for future investigation. First, the sensitivity of sequencing methodologies varies, with whole-exome sequencing demonstrating reduced sensitivity compared to targeted deep sequencing for detecting low VAF clones (<5%). Future studies should aim to optimize sequencing strategies and establish standardized thresholds for VAF in arrhythmia risk prediction. Second, more robust and diverse cohorts are needed to address the limited generalizability of current findings, which are predominantly derived from individuals of European ancestry. Expanding research to include participants from diverse racial and ethnic backgrounds would ensure broader applicability of findings and enhance understanding of CHIP prevalence and its impact across populations.

Third, the cross-sectional design of many studies precludes establishing causal relationships between CHIP and AF progression. Longitudinal studies with serial CHIP assessments are essential to determine whether the rate of clonal expansion influences arrhythmia risk and AF progression. Fourth, improved outcome ascertainment methods that minimize reliance on International Classification of Diseases (ICD) codes and self-reported data would reduce potential misclassification and enhance the accuracy of findings. Fifth, the role of myocardial fibrosis as a mediator in the CHIP-AF association requires further exploration. Future studies leveraging advanced imaging modalities such as cardiac magnetic resonance in larger and more representative cohorts are needed to confirm these relationships. Additionally, investigations into less common CHIP mutations and their specific contributions to arrhythmogenesis remain an important area for research.

Finally, while robust clinical evidence from large-scale studies supports the association between CHIP and AF, the precise pathophysiological mechanisms underlying this relationship remain incompletely understood. Current hypotheses suggest that CHIP-driven inflammation, immune dysregulation, and atrial remodeling contribute to AF pathogenesis; however, further mechanistic studies are needed to establish causality. Experimental investigations using preclinical models, single-cell transcriptomics, and functional genomic studies could provide deeper insights into how specific CHIP mutations drive atrial arrhythmogenesis.

From a translational perspective, the current evidence is largely descriptive, emphasizing the need for predictive frameworks and interventional studies. Prospective clinical trials assessing CHIP-positive individuals could help refine risk stratification strategies and evaluate targeted therapeutic approaches, such as anti-inflammatory interventions or early rhythm-control therapies. Additionally, understanding the dose-response relationship between CHIP VAF and AF progression may further aid in clinical decision-making. Future research integrating mechanistic studies with translational endpoints will be essential to bridge the gap between association and causation, ultimately informing personalized management strategies for AF in CHIP-positive individuals.

## 7. Conclusions

In summary, accumulating genetic evidence strongly supports the identification of CHIP as a novel risk factor for AF and a spectrum of other cardiac arrhythmias. These findings highlight the potential benefits of enhanced surveillance for cardiovascular health in individuals harboring CHIP mutations. Nonetheless, it is important to recognize that CHIP screening has not yet been integrated into routine cardiology practice, largely due to the lack of evidence-based interventions to address the elevated cardiovascular risk conferred by these mutations. Further research, encompassing experimental studies in animal models and clinical trials in human populations, is necessary to translate this expanding body of knowledge into targeted strategies for AF prevention and personalized management.

## Figures and Tables

**Table 1:** Summary of studies investigating the association between clonal hematopoiesis of indeterminate potential (CHIP) and atrial fibrillation (AF).

Study (Year)	Study Type	Population (n)	Main Findings	Implications
Ahn et al. [40] (2024), ‘Clonal hematopoiesis of indeterminate potential and atrial fibrillation: An east Asian cohort study’	Prospective cohort study	1004 AF patients, 3341 non-AF controls	CHIP mutations were 1.4-fold more prevalent in AF patients (23.6%) compared to non-AF subjects (10.7%). Mutations in DNMT3A, TET2, and ASXL1 were associated with more severe AF progression and a 32% increased risk of adverse outcomes.	CHIP mutations may contribute to AF development and progression, serving as potential markers for risk stratification and targets for inflammation-focused therapies.
Schuermans et al. [35] (2024), ‘Clonal hematopoiesis of indeterminate potential predicts incident cardiac arrhythmias’	Population-based cohort	410,702 participants	CHIP was associated with a 1.11-fold increased risk of supraventricular arrhythmias (HR: 1.11, 95% CI: 1.04–1.18), 1.09-fold increased risk of bradyarrhythmias (HR: 1.09, 95% CI: 1.01–1.19), and 1.16-fold increased risk of ventricular arrhythmias (HR: 1.16, 95% CI: 1.00–1.34). Large CHIP (VAF ≥ 10%) further increased these risks. TET2 mutations were strongly associated with cardiac arrest (HR: 1.81, 95% CI: 1.17–2.78) and myocardial fibrosis (OR: 1.69, 95% CI: 1.15–2.48).	CHIP is a potential age-related risk factor for arrhythmias and myocardial fibrosis, highlighting the need for further research and its potential role in precision medicine.
Lin et al. [41] (2024), ‘Clonal hematopoiesis of indeterminate potential with loss of TET2 enhances risk for atrial fibrillation through NLRP3 inflammasome activation’	Observational study and murine model	358,097 individuals, murine experiments	CHIP was associated with a 1.11-fold increased risk of AF (HR: 1.113; 95% CI: 1.044–1.187; *p* = 0.001). TET2 mutations with VAF ≥ 10% showed the strongest association (HR: 1.27; 95% CI: 1.077–1.498). Murine models revealed TET2 loss increased AF susceptibility via NLRP3 activation and calcium dysregulation in atrial cardiomyocytes.	CHIP with TET2 mutations, especially with large clones, is a strong risk factor for AF. NLRP3 inflammasome inhibitors may serve as a therapeutic strategy to mitigate AF risk in individuals with TET2 CHIP.
Saadatagah et al. [84] (2024), ‘Atrial fibrillation and clonal hematopoiesis in TET2 and ASXL1’	Population-based prospective cohort (ARIC and UK Biobank)	199,982 (4131 ARIC; 195,851 UK Biobank)	Large CHIP (HR: 1.12, 95% CI: 1.01–1.25; *p* = 0.04), large TET2 CHIP (HR: 1.29, 95% CI: 1.05–1.59; *p* = 0.02), and large ASXL1 CHIP (HR: 1.45, 95% CI: 1.02–2.07; *p* = 0.04) were associated with increased AF risk. Large TET2 CHIP correlated with elevated IL-6, and large ASXL1 CHIP with increased hs-TnT and LV mass index.	CHIP subtypes (TET2 and ASXL1) with large clone sizes are significant risk factors for AF and are linked to inflammation and cardiac remodeling. These findings support CHIP as a potential biomarker and therapeutic target for AF.
Regan et al. [85] (2025), ‘Clonal hematopoiesis associates with prevalent and incident cardiometabolic disease in high-risk individuals’	Observational cohort study (CATHGEN)	8469 participants referred for cardiac catheterization	CHIP was associated with a 1.25-fold higher odds of prevalent heart failure (HF) (adjusted OR: 1.25, 95% CI: 1.01–1.55; *p* = 0.04). Large CHIP was associated with increased risk of overall mortality (adjusted HR: 1.17, 95% CI: 1.01–1.36; *p* = 0.04). Non-DNMT3A CHIP and ASXL1 CHIP were linked to higher incident AF risk (adjusted HR for ASXL1: 2.15, 95% CI: 1.15–4.04; *p* = 0.02).	Non-DNMT3A and ASXL1 CHIP variants are key drivers of cardiometabolic risk. Findings emphasize the need for further research into specific CHIP mutations and potential interventions targeting inflammatory pathways.
Kar et al. [86] (2022), ‘Genome-wide analyses of 200,453 individuals yield new insights into the causes and consequences of clonal hematopoiesis’	Mendelian randomization	200,453 participants	CHIP was significantly associated with an increased risk of atrial fibrillation (AF) (HR: 1.09, 95% CI: 1.04–1.15, *p* = 4.9 × 10^−4^). Larger clone size further elevated AF risk, particularly with TET2 and ASXL1 mutations. Smoking and longer leukocyte telomere length were identified as causal risk factors for CHIP.	Genetic predisposition to CHIP increases AF risk, highlighting the role of clonal expansion and inflammation in arrhythmogenesis. Targeted interventions on CHIP drivers (e.g., TET2/ASXL1) and lifestyle modifications (e.g., smoking cessation) could mitigate AF risk.

Abbreviations: AF, atrial fibrillation; ASXL1, Additional sex combs-like 1; CHIP, clonal hematopoiesis of indeterminate potential; CATHGEN, Catheterization Genetics; CI, confidence interval; DNMT3A, DNA methyltransferase 3 alpha; HR, hazard ratio; hs-TnT, high-sensitivity troponin T; IL-6, interleukin-6; LV, left ventricular; NLRP3, NLR family pyrin domain containing 3; OR, odds ratio; SD, standard deviation; TET2, Tet methylcytosine dioxygenase 2; VAF, variant allele frequency.

## Data Availability

All data generated in this research are included within the article.
